# A Case of Ovariotomy

**Published:** 1872-01

**Authors:** J. S. Dolson

**Affiliations:** Hornellsville


ART. V.—*A Case of Ovariotomy. By* J. S. DOLSON, M. D.

Mrs. William Phillips, of Thurston, County of Steuben, 32 years old, married, the mother of three children, oldest thirteen, youngest four, and having six sisters and three brothers, all well; father and mother living; no hereditary disease that I could learn in the family; came under my care on the 19th day of Nov., 1871. Was in the enjoyment of good health up to about one and a half years before, and then weighed one hundred and fifteen lbs.

First discovered that the abdomen was enlarging over a year ago; menstruation regular, but somewhat less in quantity than formerly; sleeps well when the head is raised and on her side; cannot lie on her back; respiration hurried; pulse one hundred and twenty; bowels constipated; measures around body at the umbilicus forty-three inches; uterus not enlarged or displaced. I informed patient that she had an ovarian tumor, and that it was probably multilocular. As she had taken no medicine with a view to correct the growth, I advised a careful trial of the iodide of potash internally, and the application of an ointment of iodine and iodide of potash externally, saying should the treatment fail to arrest the growth it might become necessary to remove it. I visited her twice before the operation at intervals of ten days and found that she was rapidly losing flesh and strength and that the respiration was vastly worse. After detailing to her and her husband the dangers and risks of an operation, as well as I was able, and leaving them to decide what should be done, I was told by the patient she wanted even the slender chance of recovery that the operation offered.

January 8, 1872, in presence of Drs. Green, Trumbull, May and Winie, after putting patient under the influence of chloroform, I made an incision from the umbilicus to near the pubis in the linea alba, bringing tumor into view. Tapped it with a large sized trocar, with hose attached, and succeeded in removing ten lbs. of fluid. As the growth was still too large to remove through my first incision, I enlarged it upwards about two inches above the umbilicus, and after having broken down extensive adhesions with my hands, removed the mass, weighing, with the fluid previously drawn off, fifty-one lbs. The growth was largely filled with albuminous fluid of a great variety of hues, and was contained in great numbers of cysts, or what seems to me a more correct expression, compartments of different sizes, the largest one being at the upper part and in contact with the diaphram. I applied clamp and separated the pedicle with actual cautery, loosened clamp in a few moments, and, as there was still bleeding, transfixed pedicle with needle armed with double ligature, tied each part, cutting ligature close and dropped whole into abdomen. Approximated edges of wound by means of interupted sutures and straps. Applied a many tailed flannel bandage and lifted patient into bed. No stimulants were given until the operation was completed. Staid with patient about six hours; reaction has come on, patient comfortable.

Visited patient on Wednesday following operation; no vomiting and no upleasant symptoms; is taking solid opium in pill in quantities sufficient to relieve pain and produce sleep; has only a tablespoonful of milk once in two hours, and bits of ice to suck to relieve thirst.

*Hornellsville, Jan.* 11, 1872.

